# Domestication of the Dromedary Revisited and Its Consequences for Legislation as to Keeping Livestock or Pet Animals

**DOI:** 10.3390/ani13132050

**Published:** 2023-06-21

**Authors:** Marcel Smits, Han Joosten, Bernard Faye, Pamela Burger

**Affiliations:** 1 European Camel Research Society, Johanniterlaan 7, 6721 XX Bennekom, The Netherlands; 2 Microbiologist, Chemin de Crocus 1, 1073 Mollie Margot, Switzerland; 3 UMR SELMET, CIRAD-ES, Campus International de Baillarguet, 34398 Montpellier, France; 4 Research Institute of Wildlife Ecology, University of Veterinary Medicine, Savoyenstrasse 1, 1160 Vienna, Austria

**Keywords:** domestication, dromedary, house cat, legislation, keeping animals, positive list, livestock

## Abstract

**Simple Summary:**

Dutch legislators have recently introduced domestication as a requirement for animals that can be safely kept by humans and it seems likely that other countries may follow this approach. The dromedary (*Camelus dromedarius*) was considered to be insufficiently domesticated and will no longer be able to be kept in the Netherlands as livestock from 2024 onwards. In a previous scientific publication, we showed that the domestication stage of the dromedary is actually very advanced and that its evaluation by the Dutch advisory committee must be considered as a mistake. Here, we further elaborate on this topic by using the three criteria that were used by the Dutch legislators to assess the degree of domestication even though it should be noted that these criteria have neither been peer-reviewed, nor published in international scientific journals. To validate the criteria, we also assessed the domestication stage of the house cat (*Felis silvestris catus*) as this species is obviously included in the positive list. The results confirm that the domestication stage of the dromedary is very advanced. However, application of the same criteria would lead to the exclusion of the house cat. This shows that “being in an advanced stage of domestication” is not a suitable requirement for animals to be kept by humans. Instead, scientific evidence-based, peer-reviewed methodologies should be applied for legislation as to keeping livestock or pets.

**Abstract:**

Being in an advanced stage of domestication is a newly proposed requirement to decide which animals can be safely kept by humans. Dutch legislators were the first to apply it and other European countries may be tempted to adopt a similar approach. Unexpectedly, the Dutch assessors considered the dromedary (*Camelus dromedarius*) as being insufficiently domesticated and this species will therefore no longer be able to be kept as a production animal from 2024 onwards. In a recent publication on this topic, we showed that the domestication of the dromedary is actually very advanced. In this paper, we apply the same criteria that were used by the Dutch assessors to determine the degree of domestication, taking into account the most recent scientific developments in this area, even though it should be noted that these criteria have neither been peer-reviewed, nor published in an international scientific journal. For the sake of comparison, and in order to validate the procedure, we also applied these criteria to the house cat. The results confirm that the dromedary is highly domesticated, but also that the house cat (*Felis silvestris catus*) is at most semi-domesticated. Obviously, we agree with the decision of the Dutch legislators to place the house cat on the positive list, but our analysis demonstrates that this was decided on false grounds. Our analysis makes it clear that the requirement of being in an advanced stage of domestication is not suitable. Instead of maintaining this requirement, we recommend implementing evidence-based, peer-reviewed methods to decide which animals can be kept by humans, and to include species specific-guidelines in the legislation on how this can be achieved safely.

## 1. Introduction

The Dutch minister of agriculture has published a list of 30 mammal species that are allowed to be kept in the Netherlands as pets or livestock [1]. Keeping mammal species that are not on this “positive list” will be forbidden from 2024 onwards and will no longer be able to be kept as a livestock animal either. This measure was taken to avoid unnecessary suffering and health risks to both animals and humans. The dromedary was excluded from this list because an advisory committee reporting to the Dutch minister deemed it to be insufficiently domesticated, unlike, for example, the Bactrian camel (*Camelus bactrianus*), cow (*Bos taurus*), and house cat (*Felis silvestris catus*) [2].

The Dutch assessment evaluated animals at the species level. This is of course more practical from a legislative point of view, and also offers advantages for implementation and law enforcement, but inevitably reduces the possibility to fine tune requirements and approval processes as a function of breed-specific characteristics. For example, there is no specific provision for dangerous dog breeds in the national legislation and measures against animals and their owners can only be taken retroactively after bite incidents.

In several countries, legislators are regulating the keeping of animal species as pets and livestock based on a risk analysis of each species with respect to human and animal welfare [3]. The Netherlands is the first country to use “a high degree of domestication” as a requirement for admission on to the positive list and other countries may be tempted to follow this approach. However, “the degree of domestication” has never been articulated in the international scientific literature, nor has it been shown to be a useful requirement for legislative purposes.

In a previous publication, we provided numerous science-based arguments that the domestication of the dromedary is in an advanced stage [4]. Here, we assess the domestication stage of the dromedary from a slightly different angle using the same criteria that were formulated by the advisory committee that was responsible for the selection process in the Netherlands. For the sake of comparison, and in order to validate the assessment instrument, we also applied these criteria to a species that was obviously admitted on to the positive list, i.e., the house cat.

## 2. Domestication

Domestication has been defined as “a multi-generational relationship between humans and other organisms, where humans take control over their reproduction and care to have a steady supply of the organisms’ resources.” Domestication is a form of mutualism where both humans and the organisms benefit [5,6]. The first step in animal domestication is always docility, the reduced fear of humans [7]. The initial selection of tameness is characterized by a suite of morphological and physiological traits that mark the domestic state and are shared by many domesticated populations, but not by their wild ancestors: changes in coat color, floppy ears, smaller jaws and teeth, shorter reproductive cycles, and altered hormone and neurotransmitter levels, which are collectively referred as the domestication syndrome (DS) or domestication phenotype [8,9,10]. In general, two—not mutually exclusive—hypotheses have been proposed underlying the DS. The neural crest cell (NCC) hypothesis postulates that an initial selection for tameness leads to a reduced function of neural crest-derived tissues relevant for behavior via mild loss-of-function mutations in neural crest cells (NCCs). Subsequently, this neural crest hypofunction produces unselected by-products, such as morphological changes [8,9]. The thyroid hypothesis (THH) refers to an alteration in the expression of the thyroid hormone triiodothyronine (T3) and its precursor tetraiodo-thyronine (T4), which have key roles in postnatal and juvenile development, are involved in different pathways and, i.a., responsible for growth and maturation [4,8,11]. In dromedaries, genomic signals were found for both hypotheses [12]. 

**Figure 1:**
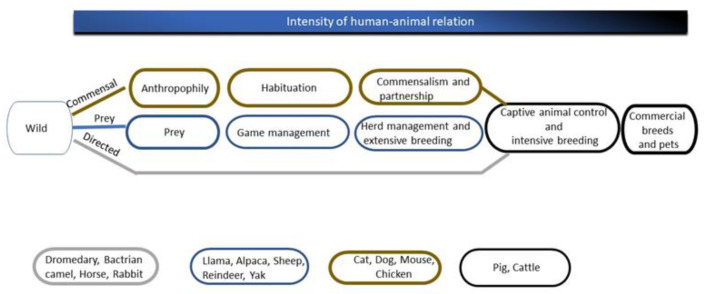
A representation of the three different domestication pathways, commensal (brown), via prey (blue) or direct (grey) including corresponding species in rectangles. On top, the increasing degree of human-animal interaction from left to right is depicted, as described by Larson and Burger (2013) [13].

As represented in Figure 1, three different domestication routes can be distinguished, which are referred to as the “commensal”, “prey” and “direct” pathways. Each of these involves captive animal control and intensive breeding, and are often followed by the development of commercial breeds and pets. Over time the intensity of the human and animal relationship increases [13]. Therefore, the Dutch advisory committee assumed that the degree of domestication relates to the manageability and welfare of the animal species. To evaluate this, the committee defined three criteria that are described in detail in Section 4. 

A clear distinction should be made between domestication and habituation. The latter involves the process of animals becoming accustomed to human presence through repeated exposure. It is a behavioral adaptation rather than a genetic one. Habituated animals gradually lose their fear response to humans and learn to tolerate their presence provided that it is perceived as non-threatening. This process is commonly observed in wildlife residing in close proximity to human settlements, where they become familiar with human activities and may even approach humans without displaying signs of fear or aggression.

Habituation is also important in domestic animals. Without habituation, domesticated animals can quickly regain several original behavioral traits of their wild ancestors. This occurs in feral domesticated animals, which have escaped from captivity or have been released [14]. The extent to which animals can become habituated to captivity varies widely between species and often also within the species. Habituation is also highly dependent on the expertise of the holder.

In summary, domestication involves the intentional modification of wild species through genetic selection, resulting in heritable traits favorable to humans. In contrast, habituation refers to a behavioral adaptation where animals become accustomed to human presence through repeated exposure, leading to a diminished fear response. While domestication alters the genetic makeup of animals over generations, habituation primarily affects an individual animal’s behavior in response to humans.

## 3. Domestication Status of the Dromedary and the House Cat

The 4000–5000-year-long domestication of the dromedary followed the “directed” pathway of domestication, i.e., targeted selection of properties useful to humans (Figure 1) [13,15,16,17,18,19,20]. Because of its naturally present properties (e.g., power and speed), initial selection was mainly exerted on tameness; although, Bedouins also bred their dromedaries specifically for velocity and milk production over many centuries [21]. Genetic ancestry background studies showed that selection for the racing ability trait from camel populations occurred over at least the past 200 years [22]. The resulting tameness after at least 4000 years of domestication [23] is anchored in the dromedary genome [12] and cannot be explained by habituation, as we showed earlier [4].

Traditionally, dromedary breeders selected their camels in the same way as breeders of other species have selected their animals for a long time, i.e., animals that did not meet the expectations of the breeders were removed from their breeding herd. Traditional breeding was based on color phenotypes, which have been related to certain economic and behavioral traits [24]. Over 200 camel populations worldwide are assigned local names. They are generically considered to be separate breeds despite the lack of a thorough examination of their ‘true’ breed status [25]. Therefore, Alaskar et al. (2021) [26] favor careful examination of the separate breeds prior to selection of specific populations for breeding/production programs and for genetic studies. Nowadays, this happens using, among others, the principles of the CD-ROM archive [27]. The late breeding age (~4 years), and the long gestation (~13 months) and weaning (~9 months) [4] as well as the pastoralist/extensive production system have prevented breeders from accurately recording the improvement of their breeds in studbooks, unlike breeders of other domesticated animals with much shorter breeding age, gestation and weaning times [27,28].

Dromedary management has changed from extensive to semi-intensive or intensive systems for breeding bulls and dairy camels and racing camels [29,30,31]. Due to recent scientific insights and technical developments, embryo transfer and artificial insemination are applied to accelerate desired commercial breeding results [32], just as has long been applied in the breeding of other commercial animal breeds. 

With respect to the house cat, archeological evidence suggests that cats have been living in close proximity of humans for 10,000 years [33,34]. Genetic exchange with populations of wildcats (*Felis silvestris silvestris*) has been very prominent and is still occurring today, which has interfered with attempts to select cats with specific desirable traits [34,35,36,37]. Contrary to other domesticated species, house cat breeds do not have different physical characteristics that facilitate executing specific tasks. Contrary to pure bred cats, commercial breeds of house cats are lacking [38]. House cats only differ from each other with respect to aesthetic differences and show very few genetic differences from their wild ancestors [35,36]. The domestication process did not have much influence on the morphological, physiological, behavioral and ecological features of cats. For this reason, there is a consensus that house cats are at most “semi-domesticated” [36,39]. 

## 4. Criteria to Evaluate the Degree of Domestication 

To evaluate the degree of domestication, three criteria have been formulated by the Dutch advisory committee. Only if each of these questions is answered with “yes”, the animal species is considered to be highly domesticated [2]. Below we answer these questions for to the dromedary and house cat.

### 4.1. Do Reliable Sources Show That Specimens of the Species in Question Are Kept by Humans?

*Explanation of the criterion:* Specimens of the species concerned are kept in man-made and controlled conditions. If animals are not kept anyway, then it can never be about animal species that are highly domesticated. In order to reach the stage in which it is possible to select animals and breed them intensively, it is absolutely necessary to keep them under controlled conditions in which they are in close contact with humans.

*Assessment of the dromedary:* Correct. Dromedaries have been kept by humans for over 4000 years [40,41,42]. In (semi-)intensive breeding systems, dromedaries are kept under controlled conditions and even in extensive production systems, where they are in close contact with humans.

*Assessment of the house cat:* Correct. Archeological evidence suggests that cats have been living in close proximity to humans for 10,000 years [33,34].

### 4.2. In the Circumstances Described, Is there Targeted, Consistent Selection and Intensive Breeding of Individuals with Human-Useful Characteristics and Traits?

*Explanation of the criterion:* Over generations, essentially similar conditions are needed because the adaptation of animals to specific conditions must also benefit future generations. To be able to select animals over generations for characteristics useful to humans, these animals must be fed, handled and cared for by humans, and the parents selected by humans for the next generations. For a population to undergo a far-reaching degree of domestication, a consistent, artificial selection will have to take place over tens of generations on human-desired traits that fundamentally change the population relative to the original species.

*Assessment of the dromedary:* Correct. Dromedaries have been kept in captivity for thousands of years and its breeding and selection has resulted in a multi-purpose animal that can be used for transport, war and as a producer of milk, meat and wool in nomadic areas and more permanent residences [23,42,43,44,45,46]. The animal of today is also characterized by an extreme tameness [27].

Nowadays, specific breeds exist that have been selected for speed (camel races) [21,47], beauty (camel beauty contests) [22,48] milk production [23], wrestling [49] and tourism [50]. To improve the distinction between breeds, genetic studbooks of different dromedary breeds are developed [27,47]. Genetic ancestry background studies have shown that selection for the racing ability trait from camel populations occurred over at least the past 200 years [22].

Artificial insemination (AI) and embryo transfer (ET) are increasingly used [23,45]. The crossing of dromedaries with two-humped Bactrian camels often happened along the Silk Roads as it combined the power of the Bactrian camel with the dromedary’s ability to adapt to cold weather [42]. This also happened in Europe [51,52]. There, too, breeding was intensive as evidenced by, for example, their slaughter for meat production in the 16th century [52]. The intensity of breeding has been further increased using AI and ET techniques.

The mere fact that the population of dromedaries that are held by humans is still increasing [24,53] also reflects the fact that its current characteristics are highly esteemed by mankind due to selection and breeding.

*Assessment of the house cat:* Incorrect. Unlike other domesticated animals, the house cat contributes little to human survival; although, they play a modest role in rodent pest control [34] and they have positive effects on human wellbeing as a companion animal. Until very recently, humans exerted hardly any control over house cats’ mate choices and living conditions, except for incidental feeding of leftovers and offering shelter. Genetic exchange with populations of wildcats may hamper attempts to select cats with specific desirable traits [34,35,36,37]. Selective breeding of cats was only initiated some 150 years ago and The International Cat Association (TICA) currently recognizes 73 standardized breeds [54]. While most other domesticated animals have different physical characteristics that allow them to perform different tasks, house cat breeds only differ from each other with respect to aesthetic differences and show very few genetic differences with their wild ancestors [35,36].

### 4.3. Has This Breeding over Generations Caused Stable Changes in Behavior, Morphology, Physiology and Reproduction in the Animal Species or Population in Question, with Which It Demonstrably Distinguishes Itself from the Original Wild Type of Individuals with Characteristics and Characteristics Useful to Humans?

*Explanation of the criterion:* It is necessary to assess the extent to which this selection at population level is reflected in differences in behavior, morphology, physiology or reproduction among the animals in the population to be assessed.

*Assessment of the dromedary:* Correct. During the 5000-year domestication process, the dromedary has become docile and highly manageable by humans. This behavior is stable [42,53]. Morphological changes have been demonstrated by comparing contemporary dromedaries with remains of dromedaries that were found at archaeological excavations at various locations in Arabia, Africa and the Near East. Bone size was significantly reduced [40]. Drawings and images have also been found of undoubtedly domesticated dromedaries [41]. Similarly, changes in skin color have occurred that changed from monochrome to mottled [40,55]. In the dromedary, positive selection has taken place on genes related to the domestication syndrome hypothesis involving both the neural crest cell (NCC) and the thyroid hormone (TH) pathways. This indicates that traits such as tameness and docility are strongly anchored in the genome of the dromedary and that physiological changes have occurred, which were not present in their wild relatives [12]. Wilkins et al. (2014) [8] have shown that the selection for tameness acts specifically on genes, which influence the formation, differentiation and migration patterns of neural crest cells. This NCC hypothesis has been confirmed in the genetic study by Rubio and Summers (2022) [56] on differences in NCC genes between 15 domesticated mammalian species, including the dromedary and their wild relatives.

*Assessment of the house cat: Incorrect.* The domestication process did not have much influence on the morphological, physiological, behavioral and ecological features of cats. For this reason, cats are considered to be “semi-domesticated” [36,39].

## 5. Discussion

Keeping animals in captivity can have many benefits for humans, but it may also cause harm to the animal and represent a hazard for its environment. It is therefore very reasonable to implement legislation defining species-specific rules and banning species that are considered to be too dangerous or that would suffer too much from being held by humans. For this reason, the Dutch minister of agriculture established a list of mammals in 2015 that could be kept in the Netherlands, but had to withdraw it following a court ruling that it lacked an objective scientific basis. Therefore, a new list was published using “a high degree of domestication” as a key requirement for admission even though this is not a scientifically recognized concept and there is no evidence that this requirement is valuable in determining whether animals can be kept as pets or livestock. In addition, the approach to determine the degree of domestication with these three questions is seriously flawed. For example, the term “intensive breeding” is not defined. We suspect that it refers to keeping a large number of animals of the same species in a restricted area just for the purpose of massive and rapid propagation, but it is not clear why intensive breeding would have any added value in this context when compared with extensive breeding systems over thousands of years. Moreover, the house cat would be condemned to being expelled from the Netherlands because this animal is certainly not the victim of “intensive breeding”. Altogether, it appears that this approach is devoid of a solid scientific basis.

The fact that dromedary camels lack the breed standard criteria and definitions that are prevalent for other livestock (Dioli 2016) [57], does not mean that commercial breeds of dromedaries are lacking and that dromedary breeders do not use artificial selection to form genetically and phenotypically distinctive populations. This might erroneously be concluded from Alaskar et al. (2021) [26], who discussed the likelihood that camel-types represent true breeds and favored careful examination of camel-types prior to the selection of populations for breeding/production studies or for genetic studies.

Larson and Burger (2013) [13] related the intensity of human–animal relationships to the extent of the domestication process. These authors concluded that the human–animal relationship is most intense at the highest degree of domestication, i.e., the formation of commercial breeds and pets. The decades-long selective and intensive breeding of dromedaries has resulted in separate commercial breeds for camel racing, beauty contests, milk production and tourism nowadays.

Nevertheless, when assessed with the three Dutch criteria, the dromedary is evidently highly domesticated and thus should have been included on the positive list.

The admission of the Bactrian camel on to the positive list while the dromedary was excluded is remarkable, knowing that the domestication process of both Old World camel species have taken a similar course [58]. In both camelids, we found a large number of genes under selection that overlap with candidate domestication genes in other domestic species [12]. The process of domestication of livestock and companion animals that started over 15,000 years ago is still ongoing and there is a surprising lack of consensus on how to define domestication [5]. Furthermore, domestication processes differ considerably between species [40]. Based on these considerations, it is evident that the “stage” of domestication is not a suitable requirement for developing legislation. This is not surprising as domestication is rather an observation tool applied to understand the roots of complex societies.

Apart from attempting to determine the degree of domestication, the advisory committee also assessed the danger of spreading zoonotic diseases of candidate species. The dromedary may carry Middle East Respiratory Syndrome Corona virus (MERS-CoV), which was considered as a “very high risk zoonosis”. However, this qualification is reserved for zoonotic diseases whose spread cannot be controlled effectively, which is not the case for MERS-CoV. Apart from the fact that this zoonosis does not occur in Europe [4], effective control measures are available that can be implemented very easily [4].

The present review shows that an objective scientific basis is lacking for the use of “highly domesticated” as a requirement to allow animal species on the positive list. If the pet and livestock list is enshrined in legislation, it is anticipated that several court cases will be initiated [59] and we expect that this review will be very useful for the lawyers to plead their case.

A literature review by Warwick and Steedman (2021) [60] identified four peer-reviewed specific methodologies for assessing the suitability of a species as a pet. However, all met scientific–political opacities and public interest requirements that can arise when governing authorities address complex issues. Therefore, the authors developed an objective positive list approach that eliminates or renders negligible a problematic consensus involvement in decision-making. Thus, the proposed method allows for a widely defensible protocol for the development of positive lists for keeping pets and livestock [60]. Consequently, we suggest that this procedure is adopted. It is to be expected that such a scientific fact-based approach will stimulate a reliable legislation to protect both human and animal welfare adequately.

## 6. Conclusions

The dromedary fulfills the requirement of being highly domesticated, while the house cat is at most semi-domesticated. Consequently, the dromedary should be admitted on to the positive list of pets and livestock and the house cat should be removed from it. As this is not a realistic scenario, it must be concluded that the requirement of being “highly domesticated” is not suitable.

Instead of pursuing this unworkable approach, we recommend implementing evidence-based, peer-reviewed methods to decide which animals can be kept by humans, and to include species-specific guidelines in the legislation on how this can be achieved safely. We would also like to encourage governments to strive for a harmonized procedure, at least in Europe, instead of attempting to address this issue with limited expertise on a national level.

## Data Availability

Not applicable.
